# Neuroperiodization After Sport-Related Concussion: A Perspective on Adaptive Load Prescription Within the Amsterdam Return-to-Sport Strategy

**DOI:** 10.3390/healthcare14172760

**Published:** 2026-09-01

**Authors:** Georgios Kakavas, Nikolaos Malliaropoulos, Jon Patricios, Florian Forelli

**Affiliations:** 1 Fysiotek Spine & Sports Lab, 17562 Athens, Greece; georgios.kakavas@gmail.com; 2 Thessaloniki Sports Medicine Clinic, 54622 Thessaloniki, Greece; contact@sportsmed.gr; 3 Sports Clinic, Rheumatology Department, Barts Health NHS Trust, London E1 1BB, UK; 4 Wits Institute for Sport and Health (WISH), Faculty of Health Sciences, University of the Witwatersrand, Johannesburg 2000, South Africa; jpat@mweb.co.za; 5 Haute-Ecole Arc Santé, HES-SO University of Applied Sciences and Arts Western Switzerland, 2800 Delémont, Switzerland; 6 Orthopaedic Surgery Department, Clinic of Domont, Ramsay Healthcare, OrthoLab, 95330 Domont, France; 7 Société Française des Masseurs—Kinésithérapeutes du Sport Lab, 95280 Asnieres sur Oise, France

**Keywords:** sport-related concussion, rehabilitation, physiotherapy, physical therapy, return to sport, exercise prescription, periodization, autoregulation, athlete monitoring

## Abstract

**Background/Objectives:** Contemporary sport-related concussion management has shifted from prolonged rest toward early, symptom-limited activity and a staged return-to-sport strategy. The Amsterdam framework provides an essential safety hierarchy but does not fully operationalize how clinicians should manipulate exercise dose, task complexity, recovery, and interacting rehabilitation domains within each stage. This Perspective proposes neuroperiodization as a constrained, criterion-based load-prescription framework nested within the Amsterdam strategy. **Framework Development:** The framework distinguishes prescribed external load from internal response, including symptom change and duration, heart rate, rating of perceived exertion, task quality, confidence, and delayed recovery. Clinical decision-making is organized through a repeated cycle of assessment, prescription, monitoring, adaptation, and reassessment. **Proposed Framework:** Neuroperiodization supports the concurrent but controlled development of aerobic, cervical, vestibular–ocular, neuromotor, cognitive–motor, and sport-specific capacities. Progression remains subordinate to medical clearance, clinical findings, symptom response, and sport-specific risk. **Conclusions:** Neuroperiodization should be regarded as a conceptual clinical framework rather than a validated rehabilitation protocol or an alternative return-to-sport clearance pathway. It integrates Amsterdam consensus recommendations, evidence-informed extrapolations, and author-proposed elements and requires population-specific adaptation and prospective evaluation.

## 1. Introduction

Sport-related concussion (SRC) rehabilitation has changed substantially over the past decade. Contemporary consensus recommendations discourage prolonged strict rest and support an early, symptom-limited return to activities of daily living and light physical activity [1]. Prescribed sub-symptom aerobic exercise can be introduced during recovery and may accelerate improvement in appropriately selected athletes [2,3,4]. Exercise is therefore no longer viewed only as a test of recovery, but also as a therapeutic intervention when appropriately selected, dosed, and monitored [1,2].

The Amsterdam return-to-sport (RTS) strategy provides an essential safety architecture by defining broad stages of activity, clarifying when head-impact risk may be reintroduced, and preserving healthcare-professional oversight and medical authorization [1]. Yet two athletes may occupy the same RTS stage while differing markedly in symptom irritability, exertional tolerance, cervical or vestibular–ocular impairments, cognitive-load tolerance, training history, sport demands, sleep, psychological readiness, and academic or occupational load [5,6]. A stage label therefore identifies what may be permissible, but it does not by itself specify the most appropriate rehabilitation dose for a particular athlete.

This distinction between stage eligibility and load prescription is directly relevant to physiotherapists. Within a single stage, clinicians must decide how much exercise to prescribe, how rapidly to progress, which domains to combine, and how to interpret mild symptom fluctuations. Evidence supports early activity and prescribed aerobic exercise [2,3,4], while targeted multimodal rehabilitation may benefit athletes with identifiable cervical, vestibular, balance, or other domain-specific impairments [5,7,8,9]. However, current evidence does not provide a universally validated algorithm for integrating all relevant load dimensions across the entire recovery pathway.

We propose neuroperiodization as a conceptual framework for addressing this operational gap. The central proposition is that the Amsterdam RTS strategy should remain the safety hierarchy, whereas neuroperiodization may function as an adaptive load-prescription overlay within that hierarchy. The framework is intended to make clinical reasoning more explicit, individualized, and testable. It is not presented as a replacement for consensus guidance, a proprietary protocol, or evidence of superiority over current care.

Existing rehabilitation periodization models were developed primarily for healthy athletic performance contexts and do not address the symptom-contingent safety constraints, multidomain heterogeneity, or staged medical authorization requirements that define SRC management [10,11]. Although periodization principles have been applied broadly in sports rehabilitation [10,11], their systematic operationalization within a concussion-specific clinical framework has not previously been attempted. Three principal insufficiencies motivate the present proposal: exertional provocation carries simultaneous therapeutic and risk implications in SRC that are absent from performance-based periodization; recovery timelines are heterogeneous and cannot be mapped to fixed calendar blocks [6]; and multiple interacting domains—aerobic, vestibular–ocular, cervical, cognitive, and psychological—require concurrent management within a medical safety hierarchy rather than a sequential performance progression [1,5,12]. Neuroperiodization is proposed as distinct from conventional rehabilitation periodization in that it is explicitly nested within a medical safety hierarchy, governed by symptom response and clinical criteria rather than time or performance targets, and designed to address multiple interacting domains concurrently. It is acknowledged that this framework was derived through conceptual synthesis, integration of existing evidence, and expert clinical reasoning rather than through a formal Delphi process, expert-panel consensus, or empirical validation study; these represent limitations of the current proposal and are addressed fully in Section 10.

The minimum features distinguishing neuroperiodization from high-quality individualized concussion rehabilitation are: (i) explicit nesting within the Amsterdam medical-authorization hierarchy rather than operating alongside it; (ii) a shared external-load/internal-response vocabulary applied consistently across rehabilitation domains rather than domain by domain; and (iii) prespecified, criterion-based progression and regression rules rather than clinician-dependent judgment alone. Individual components—individualization, progressive overload, dose–response monitoring, autoregulation, and concurrent training—are not novel; the proposed contribution is their structured integration for SRC, not the components themselves. To make this proposal testable, five explicit propositions are advanced: that structured use of the framework will improve (1) documentation completeness, (2) interprofessional communication, (3) treatment fidelity, and (4) progression-decision consistency across clinicians, and that these process improvements are hypothesized, but not yet demonstrated, to be associated with (5) more consistent symptom and functional recovery trajectories and time to unrestricted return to sport. These propositions are revisited in the Research Agenda (Section 9).

## 2. From Stage-Based Progression to Adaptive Load Prescription

Periodization is broadly understood as the planned manipulation of training variables over time to support adaptation, manage fatigue, and prepare an individual for specific performance demands [10,11]. In sports rehabilitation, it has been proposed as a bridge between impairment-focused treatment and progressive re-exposure to sport demands [10]. Its useful clinical contribution is not the imposition of rigid weekly blocks, but the deliberate organization of dose, recovery, and progression criteria [10,11].

The emerging literature has also raised concerns that residual neurocognitive and sensorimotor deficits may affect postural control, reaction time, and proprioception [13]; this is presented here as background context on residual deficits and not as part of the rationale for the framework, since an observed association between prior concussion and subsequent musculoskeletal injury does not demonstrate that neuroperiodization reduces that risk, which remains an untested hypothesis outside the scope of this proposal. Here, “neuro” is used descriptively, to refer to the injured system being rehabilitated—the central and peripheral nervous system and its sensorimotor and cognitive outputs—and not to a claimed neurobiological mechanism of action for the framework itself. No neurophysiological outcome data support the term beyond this descriptive use. Alternative, less mechanistically suggestive terms were considered; “neuroperiodization” has been retained for consistency with the title and related prior publications, with this terminological caveat made explicit.

Neuroperiodization should not be equated with a fixed five-week or five-microcycle protocol. SRC recovery trajectories are heterogeneous, making rigid calendar-based progression poorly suited to clinical practice [6]. In the proposed model, a planning block may comprise one session or several sessions and is revised whenever the athlete’s response indicates that the current prescription is insufficient, appropriate, or excessive. The clinically meaningful unit is the dose–response cycle, not the week. This dose–response cycle governs within-stage load adaptation only; it does not override the Amsterdam consensus recommendation that each formal RTS step typically requires a minimum of 24 h before advancement. Within-stage dose adjustment and between-stage progression are distinct decisions, and this distinction is maintained throughout the return-to-sport overlay presented in Section 6.

The framework distinguishes external load from internal response. External load is the work prescribed, including exercise mode, duration, intensity, work-to-rest ratio, resistance, movement speed, head movement, visual flow, surface, environmental stimulation, decision-making, dual-task demand, sport specificity, and head-impact risk. Internal response describes how the athlete responds, including symptom change and duration, heart rate, perceived exertion, movement quality, confidence, task accuracy, and delayed recovery. This distinction is established in training-load theory [14] and reduces the risk of assuming that an identical task produces an identical physiological or clinical load in every athlete (Table 1).

Several of these variables are operationally defined as follows: movement complexity is the number and unpredictability of concurrent motor demands, clinician-rated; visual flow is the density and speed of visual motion in the environment, clinician-rated; environmental stimulation is ambient noise, lighting, and crowd density, clinician-rated; task quality is accuracy or technical execution against a predefined task standard; confidence may be recorded as a pragmatic single-item 0–10 self-report for within-athlete monitoring, but this is not the I-PRRS, is not validated, and has no established cut-off; when a validated psychological-readiness measure is required, the six-item, 0–100 I-PRRS should be used [15]; and delayed recovery is time to return to pre-session symptom baseline. Only heart rate and standardized symptom scales have validated measurement instruments in this context; the remaining variables rely on structured clinician judgment, which is acknowledged as a limitation in Section 10.

For operational clarity, the following measurable anchors are proposed for clinical use. A symptom exacerbation is classified as clinically acceptable when it represents an increase of no more than 2 points on a 0–10 symptom severity scale that resolves fully within 60 min with no delayed sequelae [1]. A session is considered tolerated when no exacerbation exceeds this threshold and the athlete’s symptom rating the following morning is no greater than the pre-session baseline. A load reduction trigger is defined as any immediate symptom increase exceeding 2/10, any exacerbation persisting beyond 60 min, any delayed deterioration, or any emergent clinical sign requiring reassessment. Within-stage dose progression may occur after a single tolerated session. The two-consecutive-session criterion applies only to between-stage progression as a conservative author-proposed benchmark, with the Amsterdam minimum step duration and medical-authorization safeguards remaining applicable. These anchors are operationalized from Amsterdam consensus thresholds [1] and established load-monitoring principles [14]; they are proposed as clinical guides and have not been independently validated as progression criteria.

## 3. Boundaries of the Proposed Framework

The value of a new framework depends as much on its boundaries as on its proposed content. Neuroperiodization does not diagnose SRC, replace red-flag screening, determine independent medical clearance, or authorize exposure to contact. It does not imply that every athlete requires intervention in every domain, nor that rehabilitation must become technologically complex. It also does not convert symptoms into a precise biological “load score.” Instead, it provides a structured language for describing what was prescribed, how the athlete responded, and why the next dose was progressed, maintained, or reduced.

For athletes following an uncomplicated, rapidly resolving recovery trajectory, the framework does not mandate the full documentation set or domain-specific testing described above; routine Amsterdam-consensus care remains appropriate. Frequent symptom inquiry may itself increase symptom focus or anxiety in some athletes, and monitoring intensity should be scaled to clinical complexity rather than applied uniformly to every athlete regardless of recovery course (Table 2).

The model is therefore best viewed as a clinical reasoning scaffold. It may be particularly useful when care becomes more complex than a simple linear progression—for example, when aerobic tolerance improves but visual-motion sensitivity persists, when the athlete tolerates isolated physical and cognitive tasks but not their combination, or when clinic-based recovery is not yet matched by tolerance of school, travel, team environments, or high-speed sport demands. In these situations, a single global label such as “Step 3 completed” may conceal clinically relevant differences in exposure and readiness.

## 4. Core Principles of Neuroperiodization

### 4.1. The Amsterdam Strategy Remains the Safety Hierarchy

Neuroperiodization must be nested within the contemporary RTS strategy, not positioned as an alternative to it. Early activity and aerobic exercise can be progressed according to symptom response [1,2], whereas activities with a meaningful risk of head impact require resolution of relevant symptoms and clinical findings, successful completion of exertional stages, and authorization from an appropriately qualified healthcare professional [1]. This authorization requirement is sport- and task-dependent: it may apply from Step 3 onward whenever sport-specific exercise carries an inadvertent head-impact, collision, or fall risk (e.g., cutting sports, cycling, gymnastics), and always applies before any later activity involving such risk.

#### Symptom-Event Definitions

Four distinct events are used throughout this framework and do not carry the same clinical meaning or require the same response. Symptom exacerbation is a transient increase in existing symptoms during or shortly after activity, managed by exercise modification within the session. Symptom recurrence is the re-emergence of previously resolved symptoms, which at early, low-risk stages (Steps 1–2) may warrant exercise modification, but at later, higher-risk stages (Step 3 onward) warrants a more conservative response, up to and including regression and medical reassessment. New symptom development is the onset of a symptom not previously present and warrants session termination and clinical review. Clinical deterioration is any red-flag finding or substantial functional decline and warrants same-day or urgent medical reassessment.

Mandatory regression after mild, brief symptom exacerbation is appropriate only for the early, low-risk stages of concussion rehabilitation (Steps 1–2). The Amsterdam consensus permits a mild and brief symptom exacerbation during early activity and exercise, operationalized as an increase of no more than 2 points on a 0–10 scale for less than 1 h [1]. A larger or prolonged exacerbation should trigger cessation or reduction in the provoking load and clinical reassessment [1]. Recurrence of concussion-related symptoms during later, higher-risk stages (Step 3 onward) requires a more conservative response, up to and including regression and medical reassessment, because the consequence of error is greater.

### 4.2. Progression Is Criterion-Based, Within the Amsterdam Strategy’s Minimum Temporal and Medical Safeguards

Recovery rates vary, and a predetermined duration for each “microcycle” may either delay one athlete unnecessarily or expose another athlete to excessive demand. Progression should depend on the response to the current dose, the absence of concerning delayed effects, the quality of movement and task execution, and the clinical prerequisites for the next risk level. A planning block can be repeated, reduced, or expanded without being interpreted as failure. Criterion-based progression operates within, and never overrides, the 24 h minimum step duration and medical-authorization requirements of the Amsterdam consensus described in Section 4.1.

Criterion-based progression also requires explicit objectives. An aerobic session may aim to increase tolerated duration below a symptom threshold; a vestibular–ocular session may aim to improve gaze stability during movement; a neuromotor session may aim to restore movement quality under increasing speed; and a sport-specific session may aim to tolerate decision-making under fatigue without reintroducing head-impact risk. These objectives should not be collapsed into a single global “symptom-free” criterion.

As illustrative clinical benchmarks rather than validated cut-offs: gaze stability is judged sufficient when symptoms remain below the exacerbation threshold during standardized head–eye movement testing; movement quality is judged sufficient when a clinician-rated technical checklist for the task is met without compensation; cognitive–motor tolerance is judged sufficient when dual-task cost on a simple locomotor–cognitive pairing remains within an individually predefined threshold based on the athlete’s baseline or another specified within-athlete reference; no universal cut-off is proposed; and sport-specific decision-making is judged sufficient when reactive drills are completed at match-representative speed without a decline in accuracy or symptom exacerbation.

### 4.3. Rehabilitation Is Multidomain and Concurrent

SRC may affect several clinical domains, so an exclusively linear sequence—first aerobic work, then balance, then cognition, then sport—may be unnecessarily restrictive. Current consensus recommends multimodal assessment and targeted treatment according to the athlete’s clinical presentation [1,5,12]. Cervical and vestibular rehabilitation, prescribed aerobic exercise, and other domain-specific interventions may be indicated, although the strength of evidence and optimal timing differ across interventions and populations [5,7,8,9]. Emerging work also supports considering neuromuscular, proprioceptive, and cognitive–motor capacities when preparing athletes for high-demand sport, while acknowledging that evidence linking residual post-concussion deficits to subsequent ACL injury remains preliminary [13]. Where clinically appropriate, low-dose exposures from several relevant domains can therefore be developed concurrently, with the dominant emphasis shifting as impairments resolve and sport demands increase (Table 3).

Concurrent does not mean simultaneous maximal loading. The clinician should identify the principal stressor of each session and control the interaction among domains. For example, running while performing a complex visual–cognitive task may be appropriate later in recovery but may obscure the source of symptom provocation early on. Complexity should be added deliberately rather than as an unmeasured accumulation of demands. In practice, isolated tolerance to each relevant domain (physical, cognitive, vestibular) should be established individually before combined exposure is introduced. When a combined task provokes symptoms, the clinician should systematically reduce the task to its component parts, reintroducing one variable at a time, to identify the provoking element before resuming progression.

When multiple domain impairments are identified simultaneously, clinicians face the practical challenge of establishing rehabilitation priorities. A pragmatic hierarchy is proposed here as a clinical reasoning guide rather than a prescriptive algorithm. First priority is assigned to safety-critical presentations, including red-flag symptoms and cervical spine pathology requiring medical management before exercise progression. Second priority is given to domains that most substantially limit daily function or that present the highest symptom irritability on assessment—typically vestibular–ocular dysfunction in athletes with prominent dizziness or visual-motion sensitivity [5,9]. Once a minimum daily-functioning threshold is achieved, concurrent low-dose exposures from additional domains may begin, with one domain carrying the primary load while others are introduced at sub-threshold intensity. Interaction effects between domains should be anticipated: aerobic exercise combined with vestibular challenge amplifies the total stimulus, and fatigue from one domain may reduce tolerance in another [14]. Psychological readiness should also be monitored throughout rehabilitation, as athlete confidence and fear-avoidance responses are known to vary independently of physical symptom burden and can affect progression decisions [15]. Clinical judgment remains essential; this priority structure is not a validated decision algorithm.

### 4.4. Autoregulation Is Constrained by Clinical Safety

Autoregulation adjusts a planned prescription in response to readiness and performance. In strength and conditioning, it may use rating of perceived exertion (RPE), repetitions in reserve, movement velocity, or readiness measures [17]. In concussion rehabilitation, RPE can contribute useful information, but it cannot serve as the sole progression rule because perceived effort, symptom burden, clinical recovery, and exposure risk are related but distinct constructs.

A constrained autoregulatory approach combines RPE with symptom change, symptom duration, heart-rate response, task quality, clinical findings, and the risk associated with the activity. The athlete may adjust low-risk exercise within a predefined range, while the clinician retains control of thresholds related to rapid head movement, complex environmental exposure, high-speed sport tasks, and contact (Table 4). This preserves individualization without transferring medical clearance decisions to the athlete.

Stopping rules: activity is ceased immediately for any red-flag finding or for symptom exacerbation exceeding the threshold defined in Section 2. Contraindications to autoregulated progression include unresolved red flags, uncontrolled cervical instability, and acute vestibular crisis. Where responses across variables are discordant, symptom response is treated as the dominant safety signal and takes precedence over RPE or heart rate; deteriorating task accuracy despite stable symptoms independently warrants task modification; and low confidence despite normal clinical findings is flagged for explicit clinician discussion and possible psychological-readiness assessment [15] rather than overridden by objective findings alone.

### 4.5. Load Decisions Include Delayed Response and Total Daily Demand

The response to a session is not fully captured at session termination. Symptoms, fatigue, sleep disruption, or reduced tolerance of school and work may emerge later. Monitoring should therefore include the immediate response, recovery during the following hour, and the athlete’s status later that day or the next morning. This is especially important when several rehabilitation, academic, occupational, and social demands occur on the same day.

Each monitoring window serves a distinct purpose and is linked to a specific action: the during-session window addresses immediate safety and, if exceeded, triggers session modification; the 1 h window captures early delayed exacerbation and, if positive, triggers activity cessation for the remainder of the day; the same-day window captures cumulative load and informs next-session adjustment; and the next-morning window captures overnight recovery, including sleep-mediated effects, and can trigger medical reassessment if unresolved. Variables are integrated through a simple decision hierarchy rather than a composite score: safety-relevant findings (red flags, marked exacerbation) override all other inputs; symptom-response findings take precedence over performance or RPE findings; and performance or RPE findings inform fine-tuning only once safety and symptom criteria are already satisfied.

Sleep represents a clinically important component of total daily demand that is frequently disrupted after SRC [18]. Emerging evidence indicates that post-concussion sleep disturbance is associated with slower symptom resolution and greater daytime dysfunction, making sleep quality a relevant input variable for load adjustment alongside symptom scores, heart rate, and task performance [18]. The operating cycle should therefore routinely capture sleep quantity and quality as part of the morning reassessment step. When sleep is substantially disrupted, total rehabilitation load on the following day should be moderated even in the absence of elevated resting symptoms.

The framework discourages an overly precise composite “load number” that implies certainty unsupported by current evidence. Training-load metrics may assist description and communication, but their meaning depends on context, measurement quality, and the decision they are intended to support [14]. Recent critiques of load-management reasoning further caution against treating observational associations or arbitrary thresholds as universal causal rules [19].

### 4.6. Population-Specific Considerations

The present framework does not include population-specific algorithms, and clinicians should recognise that adaptations will be required for different athlete groups. In adolescent athletes, recovery trajectories are typically longer, cognitive-load tolerance is often reduced by concurrent academic obligations, developmental considerations affect maximal exertion testing, and sport-governing-body rules regarding return-to-play authorization frequently differ from those governing adults [1,5,6]. Sleep disruption may be proportionally more prevalent and impactful in adolescents [18]. For recreational athletes, sport-specific demand is generally lower, but occupational and social demands may substitute for competitive sport stressors and should be accounted for in total daily load estimation. Elite athletes face higher sport-specific performance demands, greater access to specialist assessment tools, and often compressed timelines due to competition scheduling, requiring particular vigilance against premature progression [5]. Para-athletes present additional complexity related to pre-existing impairment classifications, limited generalizability of current assessment normative data, and an elevated concussion symptom burden compared with able-bodied athletes [20]. Until population-specific adaptations have been systematically evaluated, the core operating principles—criterion-based progression, multidomain concurrent loading, constrained autoregulation, and delayed-response monitoring—remain applicable across groups, with individualized adjustment of specific thresholds and variables.

## 5. A Five-Step Clinical Operating Cycle

Each rehabilitation session can be organized through a repeated operating cycle. The cycle is intentionally simple so that it can be documented, communicated, and studied across settings. What makes this cycle specific to sport-related concussion, rather than a generic rehabilitation reasoning cycle, is that Assess incorporates mandatory red-flag and RTS-stage eligibility screening unique to concussion care; Prescribe and Monitor use the concussion-specific external-load and internal-response vocabulary defined in Section 2; and Adapt and Reassess apply the concussion-specific symptom-event definitions in Section 4.1, rather than generic rehabilitation criteria.

Assess: Confirm red flags (worsening headache, repeated vomiting, seizure, focal neurological signs, deteriorating consciousness, suspected cervical spine injury; see [1] for the complete criteria) and current RTS eligibility; review symptoms, sleep, fatigue, medication changes, school or work load, previous-session response, and relevant cervical, vestibular–ocular, balance, cognitive, and exertional findings. SCAT6 and SCOAT6 are assessment aids that inform, but do not independently diagnose recovery or determine return-to-sport clearance, which remains a qualified clinician’s decision. SCAT6 (or Child SCAT6 for ages 8–12) is primarily designed for acute sideline and early assessment [21], whereas office-based follow-up should use a broader multimodal assessment such as the SCOAT6 (or Child SCOAT6 for ages 8–12) and appropriate domain-specific measures [12].

Prescribe: Define the objective of the session and specify the external dose: mode, duration, intensity, work-to-rest ratio, resistance, movement speed, head movement, visual and environmental demands, cognitive–motor complexity, sport specificity, and anticipated head-impact or contact risk.

Graded exertional testing can support individualized aerobic prescription [3,4]. The Buffalo Concussion Treadmill Test (BCTT) has the stronger evidence base for determining exertional tolerance thresholds; the Buffalo Concussion Bike Test (BCBT) is a practical alternative with more limited measurement-property evidence, particularly in younger and non-ambulatory-restricted populations [22]. Contraindications include orthopedic limitations and uncontrolled cardiovascular conditions, and access to either test may be limited outside specialist settings.

The minimum documentation variables and their primary clinical purposes are summarized in Table 1.

Adapt: Progress, maintain, reduce, or stop the planned task according to predefined safety limits and the observed response. When the source of symptom provocation is unclear, adapt one or a small number of variables at a time rather than changing the entire program.

Reassess: Document recovery after the session and delayed effects. Use the combined response to determine the next dose and whether the athlete has met the clinical criteria for a higher-risk RTS stage.

**Figure 1:**
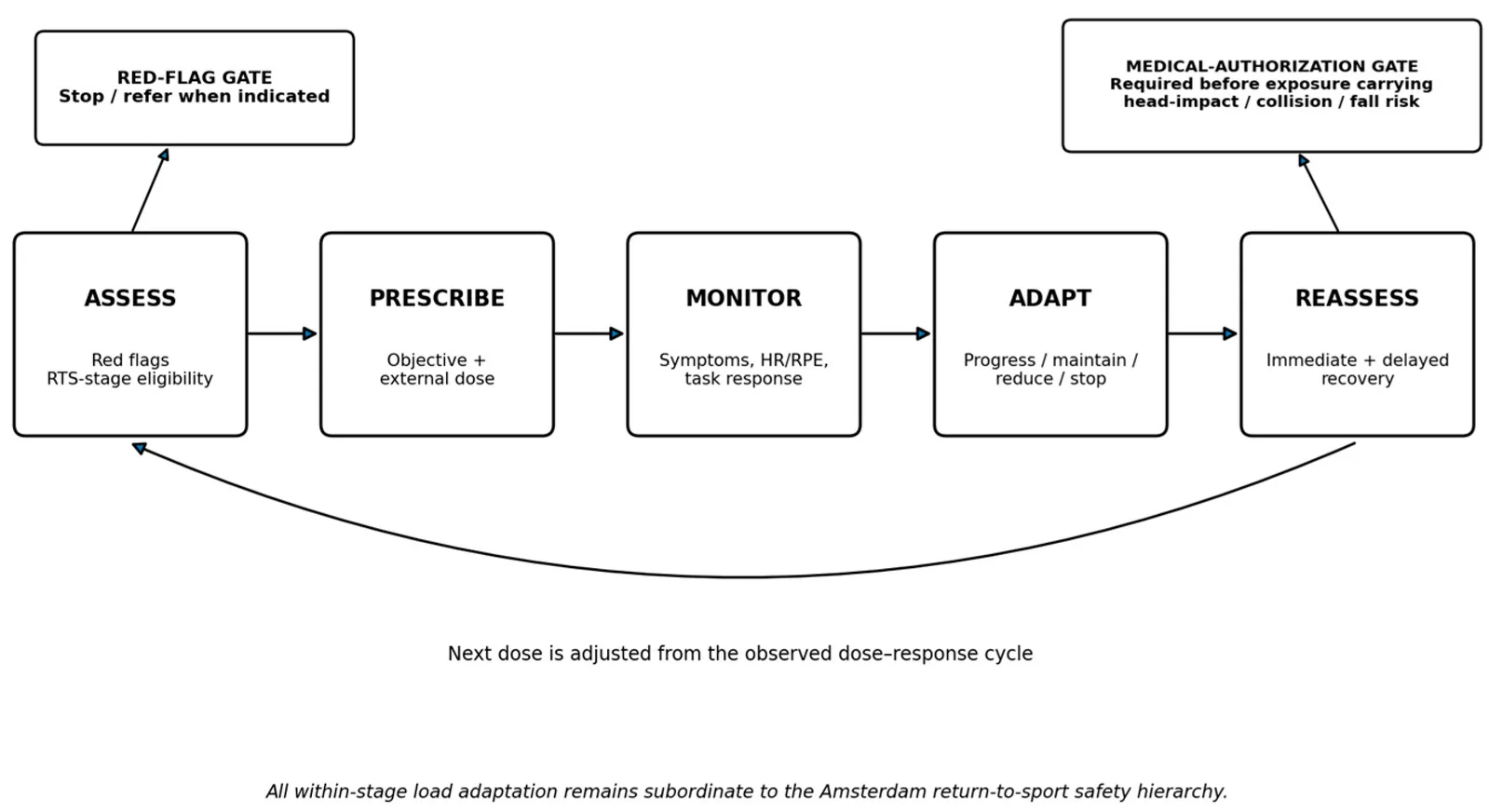
Neuroperiodization operating cycle. Red-flag screening occurs during assessment; progression to activities carrying head-impact, collision, or fall risk remains subject to the Amsterdam return-to-sport strategy and required medical authorization.

Figure 1 summarizes the assess–prescribe–monitor–adapt–reassess cycle and the safety gates that constrain progression.

## 6. Neuroperiodization Across the Return-to-Sport Pathway

Table 5 translates the proposed principles into a practical overlay. The percentages of maximum heart rate shown in the early aerobic stages are broad consensus guides rather than individualized physiological thresholds [1]. When a validated exertional test is clinically appropriate and available, a symptom-limited threshold may provide a more defensible prescription than age-predicted maximum heart rate alone [3,4,22]. The table should be read as a menu of modifiable variables and decision rules, not as a mandatory sequence of exercises. Formal exertional testing is not universally appropriate or available: contraindications, access limitations outside specialist centres, and the clinician training required for safe administration should all be considered, and symptom-limited, RPE-guided prescription remains an appropriate alternative where structured exertional testing is not accessible. Each RTS step requires a minimum of 24 h per the Amsterdam consensus [1]; the load adjustments below apply within, and do not shorten, this minimum duration. Subjective terms in the table (e.g., “stable,” “predictable”) refer to the operational anchors defined in Section 2. Age-predicted maximum heart rate (220 − age) is used by convention but has known limitations (individual variability, medication effects, autonomic dysfunction, baseline fitness, and disability); directly measured HRmax should be used when available (Table 5).

## 7. Illustrative Clinical Application

Consider a hypothetical 19-year-old association football (soccer) player assessed 8 days after SRC. For illustration: baseline symptom score is 3/10; the prescribed heart-rate range for the session is 120–140 bpm (approximately 60–70% of age-predicted HRmax, consistent with the Step 2B guide); the observed peak symptom change during the session is +1 point, resolving within 20 min; and the criterion applied for the next-session decision is whether this exacerbation remains at or below the 2-point, 60 min threshold defined in Section 2.

If symptoms increase by 1 point and resolve within 20 min without later deterioration, the next session might extend aerobic duration before increasing visual complexity. If the athlete reports greater fatigue and reduced tolerance of classes that evening, the clinician may maintain intensity but reduce density or separate aerobic and vestibular–ocular exposures across the day. Once the athlete tolerates running, change in direction, and simple ball tasks, complexity can be increased by adding visual flow or decision-making. Adding speed, dual-task demand, head movement, and environmental stimulation simultaneously would make it difficult to identify the provoking component and may create an unnecessary load spike (Table 6).

This clinical vignette is entirely hypothetical and is presented solely to illustrate the decision logic embedded in the operating cycle. It does not constitute evidence of patient outcomes, clinical effectiveness, or safety in a real-world setting. No actual patient data underpin this example, and the numerical parameters (session duration, symptom scores, RPE values) should not be interpreted as externally validated thresholds or generalizable prescriptions. Prospective implementation studies with documented patient outcomes are required before the operating cycle can be considered evidence-based.

This example is not a protocol and the numerical prescription is not intended to generalize across athletes. Its purpose is to illustrate the decision logic: identify the dominant objective, specify the modifiable variables, predefine safety boundaries, observe the immediate and delayed response, and adapt the smallest clinically relevant dimension. The framework should make the reasoning transparent without creating false precision.

## 8. Implications for Physiotherapy and Interprofessional Care

Physiotherapists are well-positioned to operationalize neuroperiodization because exercise prescription, movement assessment, cervical and vestibular rehabilitation, and graded exposure are central components of their scope in many settings [7,23,24]. Reviews support an active role for physiotherapy in SRC care [7,23,24], and a recent randomized trial found faster symptom improvement with earlier rather than later physiotherapy in a subacute mild traumatic brain injury population [8]; this evidence is indirect with respect to acute sport-related concussion and should not be extrapolated as direct support for framework effectiveness in acute SRC without further population-specific study. Earlier vestibular rehabilitation has also been associated with faster recovery after SRC in an observational study, although observational findings cannot establish causality and should also be interpreted with appropriate caution [9].

The physiotherapist’s role should remain integrated with medical diagnosis, management of red flags and comorbidities, neuropsychological or psychological input when indicated, and sport-specific decision-making by the wider performance team. Neuroperiodization may improve communication by requiring the team to describe what was loaded and how the athlete responded. “Completed Step 3” conveys less information than a record stating that the athlete tolerated 25 min of interval running, multidirectional movement, and a low-complexity visual–cognitive task at a specified RPE without more than a transient 1-point symptom increase and without delayed deterioration. Within this framework, the physiotherapist’s role is limited to load prescription and rehabilitation management; diagnosis of SRC, medical clearance, and authorization for contact exposure remain physician responsibilities. The specific division of these responsibilities varies by jurisdiction and should be confirmed against local regulation and scope-of-practice rules.

Importantly, the cognitive–motor domain within neuroperiodization addresses a residual deficit that is frequently missed by standard return-to-sport clearance procedures. Dual-task assessments have demonstrated that athletes can present with clinically meaningful cognitive–motor interference even after traditional symptom and neurocognitive measures have normalized [16]. Incorporating dual-task testing—combining locomotor and cognitive demands under progressively demanding conditions—into the monitoring phase of the operating cycle may therefore capture residual impairments that would otherwise be undetected at medical clearance. Clinicians should interpret dual-task performance in the context of the athlete’s full clinical profile rather than as a standalone criterion.

For professional education, the framework highlights the need to integrate concussion competencies with exercise physiology and load prescription. Clinicians should be able to distinguish acceptable early symptom provocation from unsafe exposure [1], understand the limits of age-predicted heart-rate targets, and use exertional testing when appropriate [3,4,22]. They should also avoid treating RPE as a substitute for clinical assessment [17]. Education should address shared decision-making, communication with coaches and schools, and jurisdiction-specific professional and legal boundaries.

For resource-limited settings, neuroperiodization should remain usable without advanced technology. A symptom scale, session duration, RPE, simple heart-rate monitoring when available, observation of task quality, and a delayed-response check may be sufficient for transparent dose adjustment. Technology can enrich monitoring but should not become a prerequisite for evidence-informed care or a source of inequity.

Using the minimum core dataset in Table 1, the estimated documentation burden is approximately 2–3 min per session. A practical communication pathway is as follows: session-level findings recorded in the clinical record; a brief plain-language summary shared with the athlete and, where relevant, parents or guardians; and periodic structured updates to coaches and schools focused on functional restrictions rather than clinical detail, consistent with confidentiality requirements.

## 9. Research Agenda

A staged development pathway is warranted: Stage 1, content validation involving athletes, parents/caregivers, coaches, physicians, physiotherapists, athletic trainers, and neuropsychologists as stakeholders; Stage 2, inter-rater reliability of the proposed criteria; Stage 3, feasibility and fidelity in real-world practice; Stage 4, safety evaluation, using a working definition of a clinically important adverse event (any symptom exacerbation exceeding the defined threshold requiring medical reassessment, or any new neurological finding during the protocol); and Stage 5, comparative-effectiveness evaluation against standard Amsterdam-consensus care.

Studies should report the exact intervention content and progression rules, because “neuroperiodization” could otherwise refer to substantially different practices. Outcomes should extend beyond days to medical clearance and include symptom burden, exertional tolerance, return to learning or work, sport-specific function, psychological readiness, adverse events, and the transition from medical clearance to sustained performance. Particular attention should be paid to whether residual cognitive–motor, proprioceptive, or neuromuscular deficits persist after symptom resolution and whether they are associated with subsequent injury; current evidence is suggestive but not sufficient to establish causality [13]. Heterogeneity by age, sex, para-sport status, symptom persistence, sport type, and access to specialist care should be planned rather than treated as an afterthought. A minimum intervention-specification checklist is recommended for any study applying the framework: the specific domains addressed, the operational load and monitoring variables used, the progression/regression criteria applied, clinician training and qualifications, and session frequency and duration, reported explicitly (e.g., as a supplementary table) so that “neuroperiodization” is not applied as a label to heterogeneous, non-comparable interventions.

Priority outcome domains for future studies should include: (i) feasibility and fidelity metrics—the proportion of sessions in which the operating cycle was completed as planned and the proportion of clinicians who can reliably apply the framework after defined training; (ii) inter-rater reliability of the proposed assessment, deficit-prioritisation, and progression criteria; (iii) safety outcomes, including the incidence and severity of symptom exacerbation episodes, the proportion of athletes exceeding predefined thresholds, and any serious adverse events; (iv) efficacy outcomes, including days to unrestricted return to sport, symptom burden trajectory, exertional tolerance threshold on standardized testing, domain-specific function scores (vestibular, cervical, cognitive–motor), and psychological readiness for return to sport assessed with validated tools [15]; (v) patient-reported outcomes, encompassing quality of life, satisfaction with care, and return to learning or work; and (vi) implementation outcomes, including documentation compliance, inter-professional communication quality, and resource utilization across practice settings. Mixed-methods designs should be used to capture quantitative clinical outcomes alongside stakeholder experiences of using the framework in real-world practice.

## 10. Limitations and Safeguards

Neuroperiodization is a conceptual synthesis derived from current concussion guidance, rehabilitation periodization, load-monitoring concepts, and autoregulation principles. It has not been prospectively validated and should not be described as an evidence-proven model. The proposed variables and operating cycle are clinically plausible, but their relative importance, thresholds, and interactions remain uncertain. The framework may also increase documentation burden if implemented without a concise workflow.

Neuroperiodization may be viewed as a structured repackaging of existing, individually validated rehabilitation principles rather than a novel intervention in its own right; the evidence supporting its components is heterogeneous, as summarized in Table 3. Increased monitoring may increase clinician documentation burden, could be inequitably accessible where resources or trained staff are limited, and could increase symptom vigilance or anxiety in some athletes.

Kakavas et al. is a narrative review authored by members of the present author group and focuses specifically on female soccer players and ACL injury risk; its citation in this manuscript has been scoped to support only that specific finding and is not used as principal support for broader cross-sport or causal claims [13]. The limitation of self-citation, and the risk of extrapolating single-sport, single-sex findings to the general SRC population addressed by this framework, is acknowledged.

Methodologically, the framework was derived through conceptual synthesis and expert clinical reasoning. No formal Delphi consensus process, structured expert-panel survey, or systematic evidence synthesis was used to generate or rank the proposed principles. The operational anchors described in Section 2—including symptom thresholds, session-tolerance criteria, and load reduction triggers—are clinically plausible and grounded in the Amsterdam consensus [1], but their inter-rater reliability, predictive validity, and optimal calibration across populations remain unknown. The illustrative clinical example in Section 7 is a hypothetical construct intended solely to demonstrate decision logic; it does not constitute evidence of patient outcomes, feasibility, or safety. Until prospective studies have evaluated the framework under real-world conditions, neuroperiodization should be treated as a structured hypothesis requiring empirical evaluation rather than an evidence-proven clinical recommendation.

The supporting literature for this Perspective was identified through a narrative, non-systematic search of PubMed and the reference lists of key consensus statements and reviews, restricted to English-language publications, without formal systematic-review methodology, dual independent screening, or a registered protocol.

Athletes who meet the referral criteria defined in Section 4.1 (symptom recurrence at a higher-risk stage, new red-flag symptom, or clinical deterioration) should undergo medical reassessment.

Several implementation challenges are anticipated. First, the operating cycle requires systematic documentation of multiple variables per session (external dose, symptom response, heart rate, RPE, task quality, delayed effects), which may increase administrative burden unless concise, standardized recording tools are developed and integrated into existing clinical workflows. Second, effective delivery of neuroperiodization requires clinicians to possess competencies spanning exercise prescription, exertional testing, vestibular and cervical assessment, and cognitive–motor evaluation; in settings where these competencies are distributed across different professionals, clear communication pathways become essential. Third, resource constraints may limit access to validated exertional testing equipment such as the Buffalo Concussion Treadmill Test [22], restricting the precision of aerobic threshold prescription in lower-resourced environments. Fourth, coordinating information among physiotherapists, physicians, neuropsychologists, coaches, and academic staff requires established interprofessional communication protocols that are not uniformly in place across healthcare systems. These implementation barriers should be explicitly addressed in feasibility research prior to wider dissemination.

Finally, periodization terminology can create an impression of precision that exceeds the evidence. Terms such as microcycle, readiness, and load should be retained only when they improve communication. The framework must remain subordinate to patient-centered clinical reasoning, shared decision-making, and the athlete’s broader life context.

## 11. Conclusions

The Amsterdam RTS strategy provides the essential safety structure for SRC recovery, but clinicians still require a transparent method for prescribing and adapting load within that structure. Neuroperiodization is proposed as a candidate framework for formal development and testing, not as an established structured language for rehabilitation. Its defining features—criterion-based progression, concurrent but controlled loading of relevant domains, separation of external dose from internal response, constrained autoregulation, and consideration of delayed recovery and total daily demand—require the staged validation outlined in Section 9 before any claim of clinical value can be made.

The proposal should be viewed as a clinically oriented hypothesis, not a validated replacement protocol. Its value will depend on whether it can be operationalized consistently, tested prospectively, and shown to improve clinically relevant outcomes without compromising safety. Until such evidence is available, neuroperiodization should be regarded as a candidate structured framework for investigation rather than an established clinical language or protocol.

## Figures and Tables

**Table 1:** Minimum core documentation dataset and primary clinical purpose.

Status	Variable	Recorded	Primary Purpose
**Mandatory**	Symptom score change (0–10 scale)	Every session	Safety monitoring
**Mandatory**	Symptom duration/time to baseline	Every session	Safety monitoring
**Mandatory**	Heart rate or RPE	Every session	Prescription/monitoring
**Mandatory**	Session-tolerance classification	Every session	Progression determination
**Mandatory**	Next-session decision (progress/maintain/regress/refer)	Every session	Progression determination
**Optional (domain-specific)**	Gaze stability, cervical range of motion, dual-task cost, etc.	When the relevant domain is targeted	Domain-specific progression determination

**Table 2:** Comparison of neuroperiodization with existing rehabilitation and periodization frameworks. Abbreviations: RPE, rating of perceived exertion; RTS, return to sport; SRC, sport-related concussion.

Feature	Traditional Sports Periodization	Multimodal Clinical-Profile Approaches	Sub-Symptom Aerobic/Graded Exposure	Rehabilitation Periodization	Amsterdam RTS Strategy	Neuroperiodization
**Primary target**	Healthy athletic performance	General musculoskeletal recovery	Individualized deficit-based treatment selection	Symptom-titrated aerobic dose escalation	SRC safety stages	SRC load prescription within safety hierarchy
**Progression basis**	Calendar and performance targets	Tissue healing and functional milestones	Clinical-profile classification and targeted treatment	Symptom-response-guided progression	Stage-based clinical criteria	Criterion-based symptom and functional response
**Safety hierarchy**	Performance risk management	Clinical oversight	Clinical oversight; not formally tied to RTS staging	Typically embedded within RTS staging	Medical authorization required at contact stages	Medical authorization required; constrained autoregulation
**Load domains**	Volume, intensity, density (physical)	Physical and functional	Domain-specific (vestibular, cervical, cognitive, etc.)	Aerobic/exertional only	Not operationally specified	Aerobic, vestibular–ocular, cervical, cognitive–motor, sport-specific
**Autoregulation**	RPE, velocity, repetitions in reserve	Limited	Clinician-directed, profile-specific	RPE/symptom-guided, single-domain	Not operationalized	Constrained by symptom response and clinical criteria
**Empirical basis**	High—extensive performance research	Moderate—clinical trials	Moderate—profile-based clinical studies	High—RCT-supported for aerobic dose [2,3,4]	High—international consensus	Conceptual; not yet prospectively validated
**Population scope**	Sport and goal specific	Injury and tissue specific	SRC; profile-dependent, not formally staged	SRC; primarily exertional-domain focus	SRC, all ages and levels	SRC; population-specific adaptations pending validation

**Table 3:** Evidentiary status of core rehabilitation components referenced in this framework.

Component	Evidentiary Status	Key Reference(s)
**Prescribed sub-symptom aerobic exercise**	Consensus-recommended; RCT-supported	[1,2,3,4]
**Amsterdam-consensus staged progression**	Consensus-recommended	[1]
**Vestibular–ocular rehabilitation**	Observational-evidence-supported	[9]
**Cervical rehabilitation**	Observational/consensus-supported	[5,7]
**Cognitive–motor dual-task training**	Extrapolated from other rehabilitation fields	[16]
**Neuromuscular/injury-prevention-oriented training**	Extrapolated; theoretical rationale	[13]

**Table 4:** Constrained-autoregulation decision matrix.

Athlete-Adjustable	Clinician-Supervised	Requires Formal Medical Authorization
**Pacing within a prescribed range; rest-interval spacing**	Session RPE target; exposure duration; combining domains within a session	Advancement to contact-risk stages; resumption of activity after a red-flag finding

**Table 5:** Neuroperiodization as an adaptive load-prescription overlay within the Amsterdam return-to-sport strategy.

RTS Phase/Clinical Intent	Dominant Objectives and Modifiable Dose Variables	Monitoring	Progression or Adaptation Rule	Evidentiary Status
Step 1 Symptom-limited daily activity	Restore daily activity and limit deconditioning without prolonged strict rest. No structured aerobic exercise is prescribed at this step; activity is guided by tolerance of usual daily tasks.	Baseline symptoms and symptom change during daily activity. Tolerance of school, work, sleep, and daily activity.	A mild, brief symptom increase (≤2/10 for <1 h) may be acceptable. Progress to Step 2A when daily activity is tolerated without exacerbation.	Consensus [1]
Step 2A Light aerobic exercise	Introduce low-risk aerobic modes once daily activity is tolerated; progress duration and frequency before complexity. Consensus guide: up to ~55% age-predicted HRmax when no individualized threshold is available. Add mobility or low-load resistance when appropriate.	Heart rate, RPE, duration, and recovery within 1 h. Delayed symptom change later that day or the next morning.	Reduce or stop if exacerbation is greater than the early-stage threshold, prolonged, or associated with concerning signs. Progress toward Step 2B when the current dose is tolerated and recovery is predictable.	Consensus [1]; RCT-supported aerobic exercise [3,4]
**Step 2B** **Moderate aerobic exercise and foundational rehabilitation**	Increase aerobic dose; consensus guide up to ~70% HRmax if no tested threshold is available. Progress resistance, cervical, balance, vestibular–ocular, and simple cognitive–motor tasks for identified impairments. Control head movement, visual flow, surface, resistance, and task density separately.	Symptoms, heart rate, session RPE, movement quality, and domain-specific findings. Delayed fatigue or reduced tolerance later that day or the next morning.	Maintain the dose if response is stable but adaptation is incomplete. Progress one or two variables after repeated tolerable exposure. Regress the provoking dimension rather than the entire program when clinically safe.	Consensus [1]; extrapolated (domain-specific components) [5,7,9]
**Step 3** **Individual sport-specific exercise without head-impact risk**	Progress locomotion, change in direction, jumping, and sport skills without head-impact risk. Increase speed, duration, density, movement variability, and controlled dual-task demand. Continue targeted care for residual cervical, vestibular–ocular, balance, or exertional impairments.	Symptoms during and after maximal non-contact exertion. Movement quality, task accuracy, confidence, RPE, heart rate, and delayed response. Clinical examination and cognitive or functional recovery as appropriate.	Progress only when symptoms and clinical findings are compatible with the next stage. A response exceeding the early-stage mild/brief threshold requires load reduction and reassessment. Do not introduce inadvertent head-impact risk. If sport-specific Step 3 exercise carries a meaningful inadvertent head-impact, collision, or fall risk, obtain the required medical authorization before exposure, consistent with Section 4.1.	Consensus [1]; extrapolated (dual-task) [16]
**Step 4** **Non-contact training drills**	Recreate high-intensity, multidirectional, reactive, cognitive sport tasks without contact. Manipulate decision uncertainty, opponent simulation, visual complexity, fatigue, and training density. Coordinate physical and cognitive demands; symptom resolution at rest alone does not establish sport readiness. Opponent-simulation and reactive drills should be explicitly risk-assessed for inadvertent collision or fall exposure before prescription, even where formal contact is prohibited.	Full-exertion symptom response, task execution under fatigue, confidence, and next-day recovery. Medical and clinical review before advancing to activities with contact risk.	Complete symptom resolution includes no symptoms during or after maximal physical and cognitive exertion. If symptoms recur, return to lower-risk non-contact exercise and reassess. Advance only after required clinical authorization.	Consensus [1]; author-proposed (risk controls)
**Step 5** **Full-contact practice**	Restore training exposure, contact confidence, tactical decisions, and team integration. Progress contact volume and density within sport rules and medical restrictions. Use neck, proprioceptive, or conditioning work for identified goals, not as proven stand-alone concussion prevention.	Symptoms, confidence, participation quality, post-practice recovery, and new clinical signs. Communication among athlete, physiotherapist, physician, coach, and relevant staff.	Formal authorization is mandatory before contact. Any recurrence of concussion-related symptoms requires removal from contact and medical reassessment. Training should demonstrate tolerance; no universal minimum number of practices is established.	Consensus [1]
**Step 6** **Return to sport/competition**	Return to competition while managing sport workload and recovery. Continue individualized care for residual non-concussion impairments when indicated. Plan the transition from medical clearance to performance restoration.	Competition response, recovery, confidence, workload, and new symptoms or injuries. Ongoing communication and access to reassessment.	Return is not the end of monitoring. New or recurrent symptoms warrant prompt reassessment. Progress workload using sport-specific conditioning principles, not a generic concussion calendar.	Author-proposed

Abbreviations: RPE, rating of perceived exertion; RTS, return to sport; SRC, sport-related concussion.

**Table 6:** Decision logic applied to the illustrative case.

Decision	Information Pattern (Using the Same Case)
**Progress**	Exacerbation within threshold (≤2/10, <60 min); rapid resolution; no delayed effects.
**Maintain**	Exacerbation at the upper limit of the threshold (≈2/10, resolving close to 60 min).
**Regress**	Exacerbation exceeding the threshold (>2/10 or >60 min) or delayed deterioration.
**Refer**	Any red-flag finding, or an exacerbation pattern inconsistent with expected recovery.

## Data Availability

No new data were created or analyzed in this study. Data sharing is not applicable to this article.

## Abbreviations

ACL: anterior cruciate ligament; BCBT: Buffalo Concussion Bike Test; BCTT: Buffalo Concussion Treadmill Test; HRmax: maximum heart rate; I-PRRS: Injury–Psychological Readiness to Return to Sport scale; mTBI: mild traumatic brain injury; RPE: rating of perceived exertion; RTS: return to sport; SCAT6: Sport Concussion Assessment Tool 6th Edition; SCOAT6: Sport Concussion Office Assessment Tool 6th Edition; SRC: sport-related concussion.

## References

[1] Patricios J.S., Schneider K.J., Dvorak J., Ahmed O.H., Blauwet C., Cantu R.C., Davis G.A., Echemendia R.J., Makdissi M., McNamee M. (2023). Consensus statement on concussion in sport: The 6th International Conference on Concussion in Sport—Amsterdam, October 2022. Br. J. Sports Med..

[2] Leddy J.J., Burma J.S., Toomey C.M., Hayden A., Davis G.A., Babl F.E., Gagnon I., Giza C.C., Kurowski B.G., Silverberg N.D. (2023). Rest and exercise early after sport-related concussion: A systematic review and meta-analysis. Br. J. Sports Med..

[3] Leddy J.J., Haider M.N., Ellis M.J., Mannix R., Darling S.R., Freitas M.S., Suffoletto H.N., Leiter J., Cordingley D.M., Willer B. (2019). Early subthreshold aerobic exercise for sport-related concussion: A randomized clinical trial. JAMA Pediatr..

[4] Leddy J.J., Master C.L., Mannix R., Wiebe D.J., Grady M.F., Meehan W.P., Storey E.P., Vernau B.T., Brown N.J., Hunt D. (2021). Early targeted heart rate aerobic exercise versus placebo stretching for sport-related concussion in adolescents: A randomized controlled trial. Lancet Child Adolesc. Health.

[5] Schneider K.J., Critchley M.L., Anderson V., Davis G.A., Debert C.T., Feddermann-Demont N., Gagnon I., Guskiewicz K.M., Hayden K.A., Herring S. (2023). Targeted interventions and their effect on recovery in children, adolescents and adults who have sustained a sport-related concussion: A systematic review. Br. J. Sports Med..

[6] Putukian M., Purcell L., Schneider K.J., Black A.M., Burma J.S., Chandran A., Boltz A., Master C.L., Register-Mihalik J.K., Anderson V. (2023). Clinical recovery from concussion—Return to school and sport: A systematic review and meta-analysis. Br. J. Sports Med..

[7] Art K., Ridenour C., Durbin S., Bauer M., Hassen-Miller A. (2023). The effectiveness of physical therapy interventions for athletes post-concussion: A systematic review. Int. J. Sports Phys. Ther..

[8] Campbell K.R., Antonellis P., Peterka R.J., Wilhelm J.L., Scanlan K.T., Pettigrew N.C., Chen S., Parrington L., Fino P.C., Chesnutt J.C. (2025). In people with subacute mild traumatic brain injury, earlier physical therapy improved symptoms at a faster rate than later physical therapy: Randomized controlled trial. Phys. Ther..

[9] Anderson M., McCorkle M., Hammonds K., Reynolds E., Gilliland T., Covert K., Driver S. (2025). Early vestibular rehabilitation initiation is associated with faster recovery after sport related concussion. J. Sci. Med. Sport.

[10] Lorenz D.S., Reiman M.P., Walker J.C. (2010). Periodization: Current review and suggested implementation for athletic rehabilitation. Sports Health.

[11] Lorenz D., Morrison S. (2015). Current concepts in periodization of strength and conditioning for the sports physical therapist. Int. J. Sports Phys. Ther..

[12] Patricios J.S., Davis G.A., Ahmed O.H., Blauwet C., Schneider G.M., Purcell L.K., Echemendia R.J., Fremont P., Fuller G.W., Herring S.A. (2023). Introducing the Sport Concussion Office Assessment Tool 6 (SCOAT6). Br. J. Sports Med..

[13] Kakavas G., Malliaropoulos N., Skarpas G., Forelli F. (2025). The Impact of Concussions on Neuromuscular Control and Anterior Cruciate Ligament Injury Risk in Female Soccer Players: Mechanisms and Prevention—A Narrative Review. J. Clin. Med..

[14] Impellizzeri F.M., Marcora S.M., Coutts A.J. (2019). Internal and external training load: 15 years on. Int. J. Sports Physiol. Perform..

[15] Crofts R., Morris A.J., Quammen D.L., Petersell T.L., Liebel S.W., Podlog L., Fino P.C. (2025). Confidence to return to play after concussion. J. Sport Rehabil..

[16] Hashida K., Shirahata K., Furutani T., Tamura K. (2024). Clinically feasible dual-task detects residual post-concussion deficits after return-to-play. J. Sports Sci..

[17] Greig L., Stephens Hemingway B.H., Aspe R.R., Cooper K., Comfort P., Swinton P.A. (2020). Autoregulation in resistance training: Addressing the inconsistencies. Sports Med..

[18] Donahue C.C., Resch J.E. (2024). Concussion and the Sleeping Brain. Sports Med. Open.

[19] West S., Shrier I., Impellizzeri F.M., Clubb J., Ward P., Bullock G. (2025). Training-load management ambiguities and weak logic: Creating potential consequences in sport training and performance. Int. J. Sports Physiol. Perform..

[20] Ryan T., Ryan L., Daly E. (2024). Concussion in parasport: A narrative review of research published since the Concussion in Para Sport (CIPS) Group Statement (2021). Healthcare.

[21] Echemendia R.J., Burma J.S., Bruce J.M., Davis G.A., Giza C.C., Guskiewicz K.M., Naidu D., Black A.M., Broglio S., Kemp S. (2023). Acute evaluation of sport-related concussion and implications for the Sport Concussion Assessment Tool (SCAT6) for adults, adolescents and children: A systematic review. Br. J. Sports Med..

[22] Janssen A., Pope R., Rando N. (2022). Clinical application of the Buffalo Concussion Treadmill Test and the Buffalo Concussion Bike Test: A systematic review. J. Concussion.

[23] Robertson M.K., McLoughlin J. (2024). The role of the physiotherapist in concussion. S. Afr. J. Physiother..

[24] Bishay A.E., Godwin S.L., Jo J., Williams K.L., Terry D.P., Zuckerman S.L. (2025). The role and benefits of physical therapy following sport-related concussions. J. Sport Rehabil..

